# Analysis of host factors for internal ribosomal entry site-mediated translation of foot and mouth disease virus and classical swine fever virus: an *in vivo* evaluation in single- and double-knockout mice

**DOI:** 10.1128/jvi.00905-26

**Published:** 2026-07-22

**Authors:** Masashi Utsunomiya, Kenki Momohara, Risa Sato, Suzune Nagatomo, Tatsuro Hifumi, Yoshihiro Sakoda, Kyoko Tsukiyama-Kohara

**Affiliations:** 1 Joint Faculty of Veterinary Medicine, Transboundary Animal Diseases Center, Kagoshima University 12851, Kagoshima, Japan; 2 Department of Veterinary Pathology, Joint Faculty of Veterinary Medicine, Kagoshima University 12851, Kagoshima, Japan; 3 Department of Veterinary Microbiology, Faculty of Veterinary Medicine, Hokkaido University 12810, Sapporo, Japan; St Jude Children's Research Hospital, Memphis, Tennessee, USA

**Keywords:** PKD1L3, USP31, FMDV IRES, CSFV IRES, CSFV replicon, EMCV infection

## Abstract

Foot-and-mouth disease virus (FMDV) and classical swine fever virus (CSFV) both exploit host factors for internal ribosomal entry site (IRES)-mediated translation when they infect hosts. We previously identified polycystic kidney disease 1-like 3 (PKD1L3) and ubiquitin-specific peptidase 31 (USP31) as host factors for IRES-mediated translation, common to both viruses *in vitro*. To further investigate these host factor roles in IRES activity and viral replication *in vivo*, we established *Pkd1l3*^−/−^, *Usp31^−/−^*, and *Pkd1l3^−/−^Usp31^−/−^* (double-knockout [DKO]) mice. These knockout mice were viable through 1 year after birth. FMDV and encephalomyocarditis virus (EMCV) IRES activities were significantly suppressed in *Pkd1l3^−/−^*, *Usp31^−/−^*, and DKO mouse liver tissue. CSFV IRES activity was decreased, but non-significantly, in single-knockout and DKO mouse liver tissue. CSFV replicon replication was significantly decreased in the livers of USP31^−/−^ and DKO mice. Mice were further infected with porcine EMCV (an FMDV surrogate), and *Pkd1l3*^−/−^, *Usp*31^−/−^, and DKO mice showed slightly less neuronal ischemic necrosis in the cerebral cortex than wild-type (WT) mice. Extramedullary hematopoiesis in the spleen was observed only in WT cases. Viral titer and/or RNA level was significantly decreased in the lung in all KO mice, and in the brain in DKO mice. In the heart, a major target for this virus, copy number levels decreased in the order of WT, *Pkd1l3^−/−^*, *Usp31^−/−^*, and DKO, albeit without showing significant differences. Accordingly, this study provides useful evidence on PKD1L3 and USP31 host-factor deletion, as a step to developing FMDV- and CSFV-resistant livestock through the prevention of IRES-mediated translation.

**IMPORTANCE:**

Foot-and-mouth disease virus (FMDV) and classical swine fever virus (CSFV) are highly infectious to livestock, and novel mitigation strategies are required. Establishment of FMDV- and CSFV-resistant animals has the potential to prevent FMD and CSF infection. This study provides useful evidence for the development of such resistant livestock, through an *in vivo* evaluation of host factors for internal ribosomal entry site (IRES)-mediated translation.

## INTRODUCTION

Foot-and-mouth disease virus (FMDV) is an aphthovirus in the *Picornaviridae* family, and classical swine fever virus (CSFV) is a pestivirus in the *Flaviviridae* family. Both viruses are major pathogens of livestock (1, 2). FMDV primarily affects cloven-hoofed mammals and causes vesicular lesions in the oral cavity and on the feet of infected animals. CSFV affects wild and domestic pigs and causes symptoms, including fever, diarrhea, and neurological signs, and is associated with high mortality rates. Vaccination and culling are among the measures used to control these pathogens; however, these approaches can be costly and challenging. FMDV currently has six circulating serotypes (3), and multi-serotype vaccines are not available. Furthermore, FMDV vaccines may not protect against subclinical forms of the disease. Vaccination efforts to control CSFV infections have been relatively successful, although recent outbreaks have been reported in previously vaccinated pigs in China (1). Accordingly, there is a need for additional mitigation strategies, and disease-resistant livestock have the potential to represent one such novel mitigation strategy. However, the development of disease-resistant animals depends on an enhanced understanding of virus biology and the mechanisms by which FMDV and CSFV infect hosts.

FMDV and CSFV both possess single-stranded, positive-sense RNA genomes, and translation into a virus-encoded polyprotein is initiated from an internal ribosomal entry site (IRES) within the 5′-untranslated region (5′UTR) (4, 5). Both viral and cellular IRESs adopt unique higher-order structures and require distinct eukaryotic initiation factor (eIF) or host factor interactions, which make IRES-mediated translation distinct from cap-dependent translation (6). IRESs can be classified into five groups (2): the FMDV IRES belongs to Group I (the picornavirus group), and the CSFV IRES belongs to Group IV (the hepatitis C virus group) (7). Although FMDV and CSFV belong to different virus families and possess IRESs in different groups, we previously identified two host factors that may participate in IRES-mediated translation for both viruses, suggesting the possibility of common mitigation strategies. These two factors are polycystic kidney disease 1-like 3 (PKD1L3) and ubiquitin-specific peptidase 31 (USP31), and we found their expression in IRES-expressing cells was reduced in microarray experiments following treatment with the antiviral agent pycnogenol, and silencing of the relevant encoding genes (8). In a subsequent *in vitro* study, we then substantiated the roles of PKD1L3 and USP31 as common host factors for FMDV and CSFV IRES-mediated translation, based on significantly decreased IRES activity following gene silencing, immunoprecipitation assays demonstrating that the two proteins interact with each other and with eIF3c, and immunofluorescence assays demonstrating cytoplasmic colocalization (9). These findings suggest that knockdown of *Pkd1l3* and *Usp31* may be a promising strategy for developing disease-resistant livestock. However, these previous results were all obtained from *in vitro* studies, and evidence obtained from an *in vivo* study is required to advance this research.

The mouse is an ideal model for initial *in vivo* studies of PKD1L3 and USP31 host factor deletion. Mice offer many advantages, including low cost, ease of handling, and their suitability for gene editing. In addition, mice are reportedly susceptible to a picornavirus, which can be used as a surrogate for FDMV where use of the latter in experimental infection is not permitted. This is porcine encephalomyocarditis virus (EMCV), which possesses an IRES in the same group as FMDV, and is known to cause disease in mice, either a lethal infection in BALB/c mice (3) or largely non-lethal infections with milder pathology in C57BL6/J mice (10, 11). Thus, the C57BL6/J mouse may represent a useful strain to evaluate gene knockout (KO) *in vivo*, as the evaluation may be unaffected by acute death.

Against this background, in the present study, we set out to establish single- and double-knockout mice for the deletion of the host factors PKD1L3 and USP31, and to then evaluate the effects on IRES activities, and FMDV and CSFV infection in *Pkd1l3*^−/−^ and *Usp31*^−/−^ single-knockout mice and double-knockout (DKO) mice, as an initial step for *in vivo* research.

## RESULTS

### Establishment of single-knockout (Pkd1l3^−/−^ and Usp31^−/−^) mice

**Fig 1:**
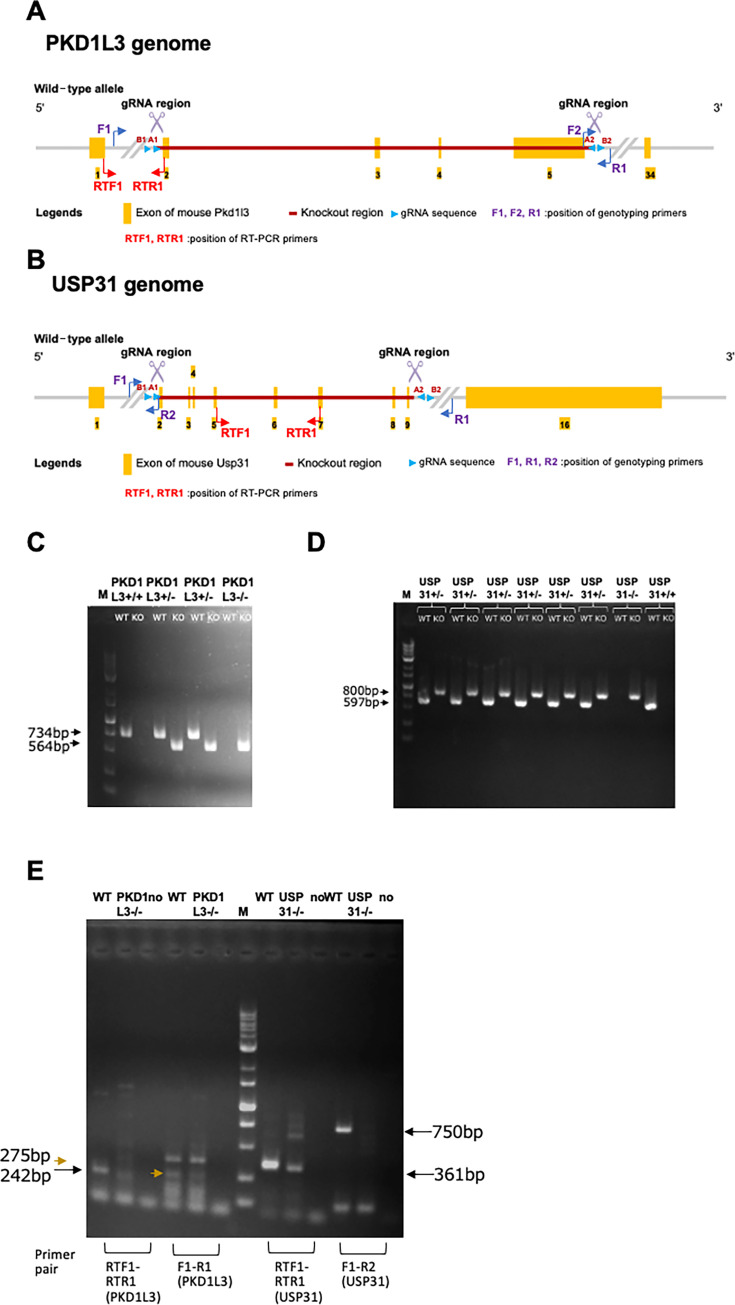
(**A**) Targeting of *Pkd1l3* in the mouse genome. Exons 1–34 are indicated. The genomic region targeted for knockout by Crispr/Cas9 editing is indicated with a red line surrounded by gRNA sequences B1, A1, A2, and B2. The positions of primers for genotyping (F1, F2, and R1) and RT-PCR (RTF1 and RTR1) are also shown. (**B**) Targeting of *Usp31* in the mouse genome. Exons 1–16 are indicated. The genomic region targeted for knockout by Crispr/Cas9 editing is indicated with a red line surrounded by gRNA sequences B1, A1, A2, and B2. The positions of primers for genotyping (F1, R1, and R2) and RT-PCR (RTF1 and RTR1) are also shown. (**C**) PCR genotyping for *Pkd1l3*. The KO genome was detected as bands with 564 bp, and the WT genome was detected as bands with 734 bp. (**D**) PCR genotyping for *Usp31*. The KO genome was detected as bands with 800 bp, and the WT genome was detected as bands with 597 bp. (**E**) Detection of *Pkd1l3* mRNA expression in WT and *Pkd1l3^−/−^* mice (left) and *Usp31* mRNA expression in WT and *Usp31*^−/−^ mice (right) by RT-PCR. Reactions without mouse genomic DNA are indicated as “no.”

To generate single-knockout mice lacking the PKD1L3 or USP31 host factor for IRES-mediated translation *in vivo*, we crossbred F0 heterozygous mice and evaluated their offspring with PCR-based genotyping and RT-PCR assays for RNA expression. The F0 heterozygous mice were obtained from the supplier after deletion of the *Pkd1l3* or *Usp31* genomic region on a C57BL6/J genetic background by the CRISPR/Cas9 method. That procedure involved removal of 9,477 bp or 13,203 bp surrounded by gRNA sequences, including exons 2–5 (Fig. 1A), or 2–9 (Fig. 1B), as described in Materials and Methods.

In genotyping, bands of the expected size were observed for F1 WT and homozygous knockout mice (*n* > 3, for each type). Representative examples of bands as visualized after electrophoresis are shown in Fig. 1C and D. In the RT-PCR assay, we confirmed the absence or a different size with less expression of detectable bands of the expected size for the deleted genes (Fig. 1E). The genotype distributions for the F1 single-knockout mice from three heterozygous knockout parent pairs for *PKD1L3* and *USP31* were as follows. In the evaluation of PKD1L3 deletion, 4% (1/25, 1 male) were wild-type (*Pkd1l3*^+/+^) mice, 44% (11/25, 6 males, 5 females) were heterozygous knockout mice (*Pkd1l3*^+/−^), and 52% (13/25, 3 males, 10 females) were homozygous knockout mice (*Pkd1l3*^−/−^). The corresponding figures in the evaluation for deletion of the *Usp31* gene were 33.3% (7/21, 5 males, 2 females) for wild type (*Usp31*^+/+^), 57.1% (12/21, 7 males, 5 females) for heterozygous knockout mice (*Usp31*^+/−^), and 9.5% (2/21, 1 male, 1 female) for homozygous knockout mice (*Usp31*^−/−^). These results showed that *Pkd1l3*^−/−^ mice appeared more frequently, especially among females, and *Usp31*^−/−^ mice less frequently than would be expected for true Mendelian inheritance.

### Establishment of DKO mice and genotype comparisons

To establish DKO mice for further evaluation of the PKD1L3 and USP31 host factors, we crossbred *Pkd1l3*^+/−^ *Usp31*^−/−^ and *PKD1L3^−/−^Usp31*^+/−^ mice and evaluated their offspring with PCR-based genotyping and for body weight and viability in comparisons with single-knockout and WT mice, through a 28-day period after birth, and at 1 year after birth.

**Fig 2:**
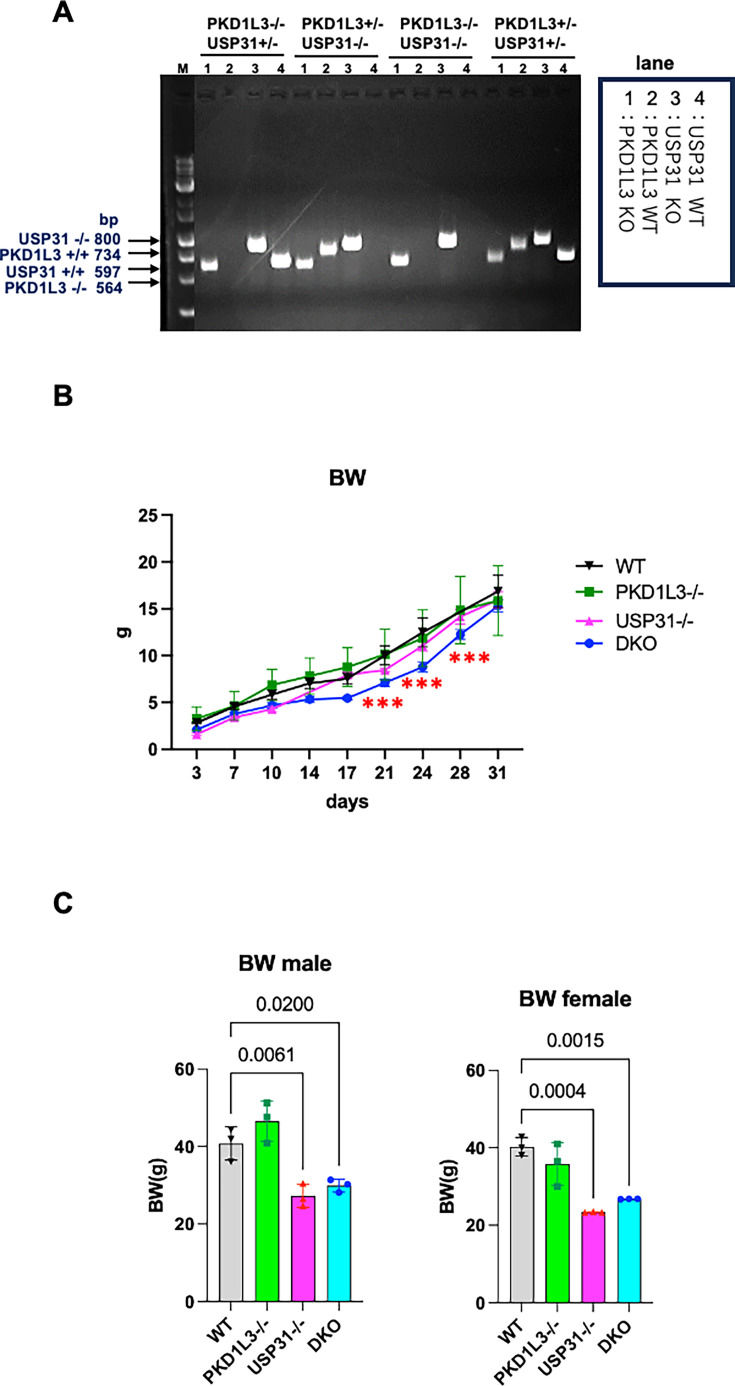
(**A**) Establishment of the DKO mouse by crossbreeding and genotyping. All mice shown here are female. (**B**) Transition in body weight for WT (*n* = 9, 5 males, 4 females)*, Pkd1l3*^−/−^ (*n* = 7, 3 males, 4 females), *Usp*31^−/−^ (*n* = 5, 4 males, 1 female), and DKO mice (*n* = 9, 6 males, 3 females) from birth. Statistical analysis was performed at each time point with one-way ANOVA and Dunnett analysis, and statistically significant points are indicated (***P < 0.001). (**C**) Mean body weight in 1-year-old male (left) and female (right) mice from WT, *Pkd1l3*^−/−^, *Usp*31^−/−^, and DKO mice (*n* = 3, for each type). Vertical bars indicate standard deviation (S.D.). *P* values are indicated, and a value less than 0.05 is significant.

Genotyping revealed that 33.3% of F1 offspring were homozygous DKO mice (*Pkd1l3*^−/−^, *Usp31*^−/−^). Representative examples of bands as visualized after electrophoresis are shown in Fig. 2A.

There were no differences in viability between the genotypes through the 28-day period after birth, or at 1 year after birth.

Body weight was significantly lower in DKO than WT mice from day 21 after birth through day 28 after birth (Fig. 2B). At 1 year after birth, body weight was significantly lower in *Usp31*^−/−^ (*P* = 0.0061/.0004; male/female) and DKO (*P* = 0.02/0.0015; male/female) than WT mice for both sexes (Fig. 2C). No other significant differences in body weight between genotypes were observed.

### Effect of Pkd1l3 and Usp31 knockout on IRES activities *in vivo*

To clarify the roles of PKD1L3 and USP31 as host factors for IRES-mediated translation *in vivo*, we transfected bicistronic vectors containing FMDV (pRF) and CSFV (pCI5) IRESs (12) into 10-month-old male single-knockout (both *Pkd1l3*^−/−^ and *Usp31*^−/−^), DKO (*Pkd1l3*^−/−^ *Usp31*^−/−^), and WT mice and compared the IRES activities between the genotypes.

**Fig 3:**
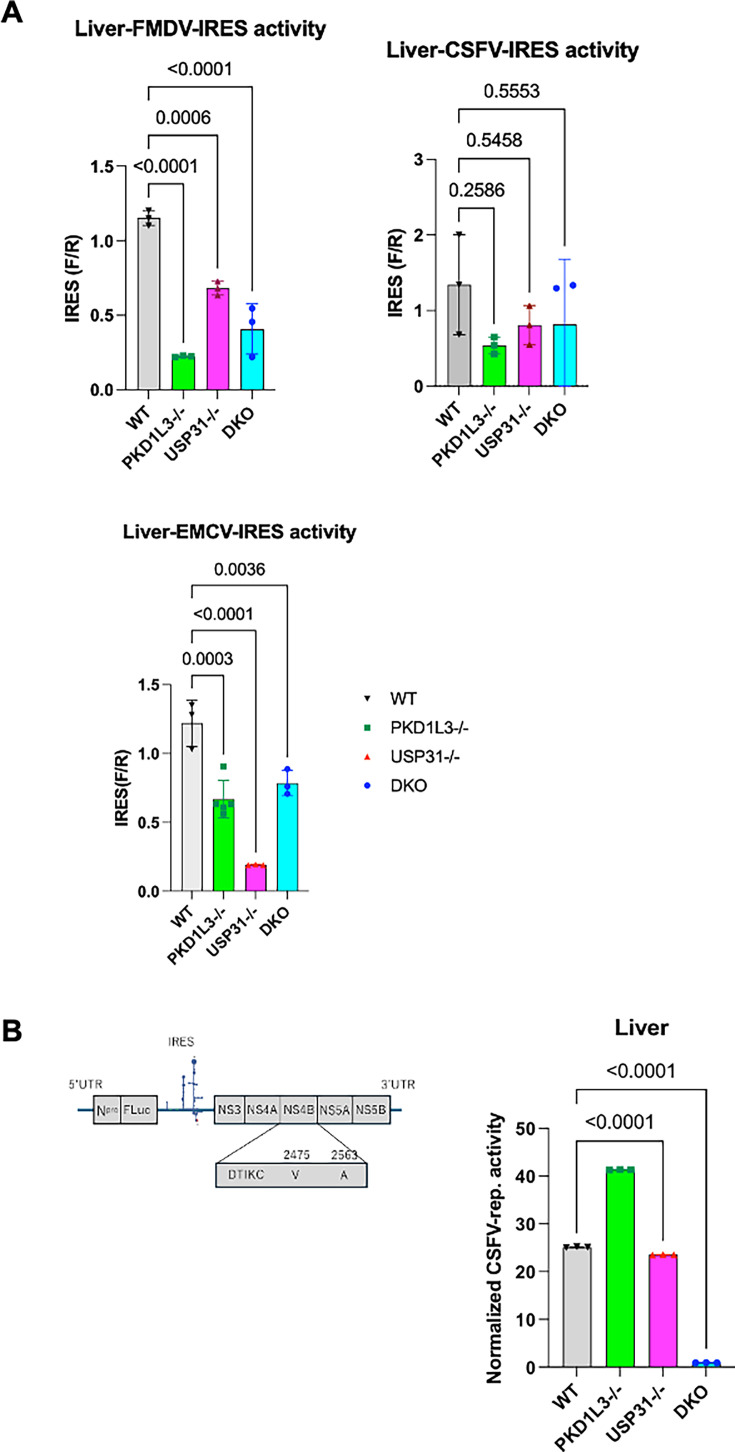
(**A**) Detection of IRES activities from the bicistronic FMDV-IRES mRNA (left), CSFV-IRES mRNA (right), and EMCV-IRES mRNA (lower) in liver tissue from WT, *Pkd1l3*^−/−^, *Usp*31^−/−^, and DKO mice (*n* = 3, respectively). *P* values are indicated, and a value less than 0.05 is significant. (**B**) CSFV-replicon structure is indicated (left), and CSFV replication is represented by the measured FLU activity in mouse liver lysate normalized with protein concentration (μg/mL) and GAPDH (copies/mL), for WT, *Pkd1l3*^−/−^, *Usp*31^−/−^, and DKO mice (right) (*n* = 3, respectively). Vertical bars indicate standard deviation (S.D.). *P* values are indicated, and a value less than 0.05 is significant.

FMDV and EMCV IRES activities were significantly suppressed in both *Pkd1l3*^−/−^ and *Usp31*^−/−^ mice and DKO mice when compared with WT mice (*P* < 0.0001–0.0036; Fig. 3A, left and lower). CSFV IRES activity showed no significant difference, although the values for the three knockout genotypes were lower than those observed for WT mice (Fig. 3A, right and lower).

### Effects of PKD1L3 and USP31 knockout on CSFV replication *in vivo*

To clarify the role of PKD1L3 and USP31 in CSFV replication, we injected a CSFV replicon RNA harboring a reporter gene (rGPE-N^pro^-Luc-IRES-NS3) *in vivo* (Fig. 3B) and evaluated replication as luciferase activity in the liver normalized to protein concentration and GAPDH copy number. CSFV replication was significantly reduced in *Usp31*^−/−^ (*P* = 0.0002) and DKO (*P* = 0.0001) mice versus WT mice. The reduction was more pronounced in the DKO mice (Fig. 3C).

### Effects of PKD1L3 and USP31 deletion on viral infection *in vivo*

**Fig 4:**
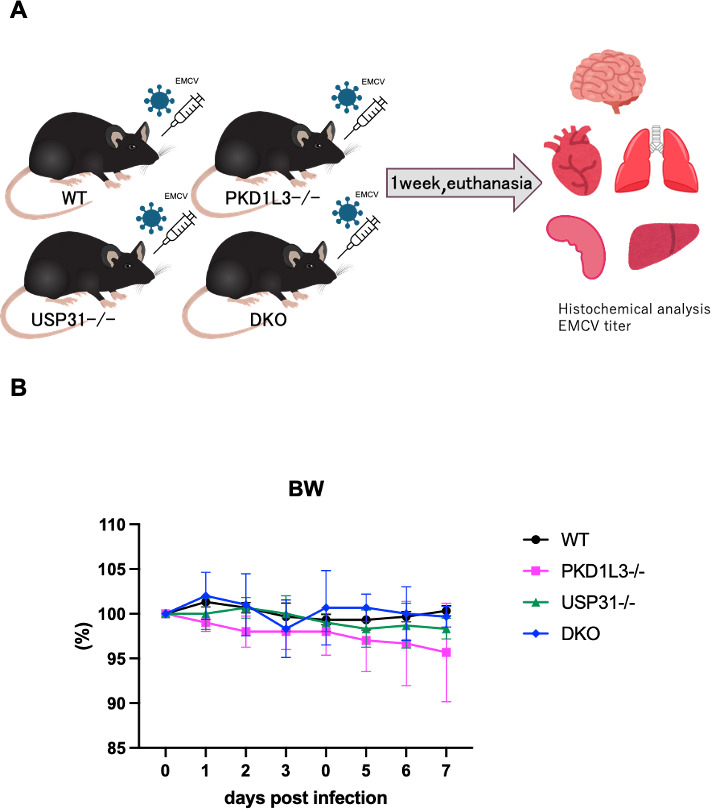
(**A**) Schematic diagram of the experiment. WT, *Pkd1l3*^−/−^, *Usp*31^−/−^, and DKO mice (*n* = 3, respectively; male ratio in each group ranges from 0% to 66%) were inoculated (ip) with EMCV (4 × 10^5^ TCID_50_), and tissues were collected after sacrifice at 1 week post-inoculation for histopathological evaluation. (**B**) BW change ratio (%) is shown for WT, *Pkd1l3*^−/−^, *Usp*31^−/−^, and DKO mice inoculated with EMCV over a 1-week period. Vertical bars indicate S.D.

**Fig 5:**
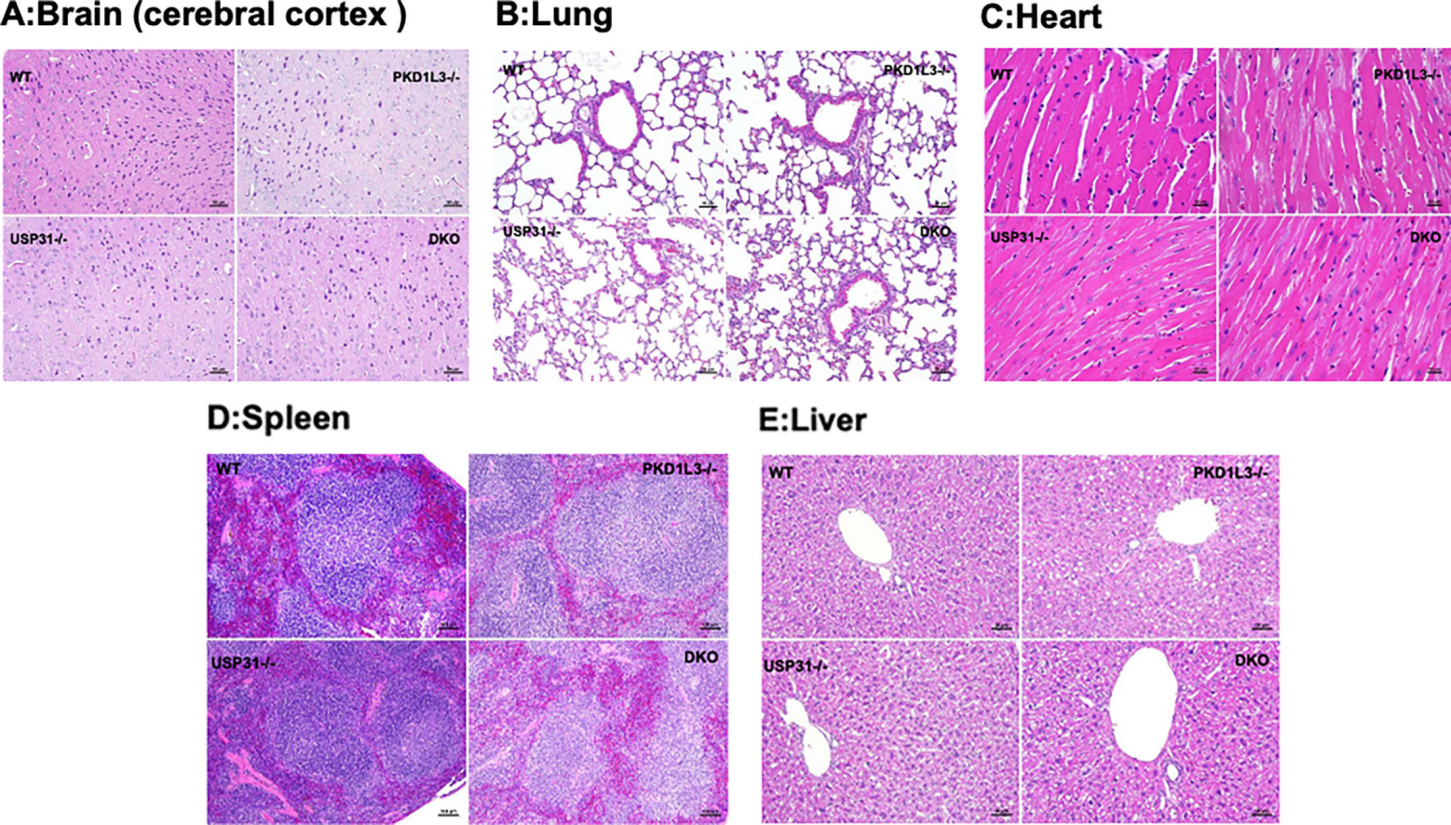
Histopathological images (hematoxylin and eosin staining) of mouse cerebral cortices (**A**), lungs (**B**), hearts (**C**), spleens (**D**), and livers (**E**) from WT, *Pkd1l3*^−/−^, *Usp*31^−/−^, and DKO mice inoculated with EMCV (bars = 20–100 µm) (*n* = 3, representative images are shown).

To clarify the role of PKD1L3 and USP31 in picornavirus infection *in vivo*, we inoculated 10-month-old single-knockout (*Pkd1l3*^−/−^ or *Usp31*^−/−^), double-knockout (*Pkd1l3*^−/−^, *Usp31*^−/−^), and WT mice, and evaluated them for body weight and histopathological lesions. EMCV is a picornavirus that possesses a type II IRES, the same group as the FMDV IRES (13), and is known to cause disease in mice (3). Thus, EMCV is a good candidate to model FMDV-like IRES-mediated translation, given that the experimental use of FMDV is not approved in Japan. The mice were infected with EMCV intraperitoneally at 4 × 10^5^ TCID_50_, as previously reported (3). The mice were sacrificed under anesthesia at 7 days after EMCV infection, and we collected the lungs, heart, liver, spleen, and brain from each mouse for histopathology and RNA quantification (Fig. 4A), as the most relevant previous reports involved setting 7 days after EMCV infection as the endpoint (3, 10, 14). Body weight was maximally decreased in *PKD1L3*^−/−^ mice, but this decrease was not significant when the value was compared with that in WT in the first week post-infection (Fig. 4B). There were no other significant abnormalities in EMCV-infected mice, including in behavior and body temperature. Histopathological findings were as follows. Single-knockout and DKO mouse brains showed slightly less neuronal ischemic necrosis than WT mouse cerebral cortices (Fig. 5A). Extramedullary hematopoiesis in the spleen was observed only in WT cases (Fig. 5D). Mild fatty degeneration of hepatocytes was observed in the livers of *PKD1L3*^−/−^ mice (Fig. 5E). All mice (of all genotypes) showed hemorrhage and congestion in the red pulp region of the spleen (Fig. 5D). The single-knockout and DKO mice showed no marked differences from WT mice in the lungs (Fig. 5B) or heart (Fig. 5C).

**Fig 6:**
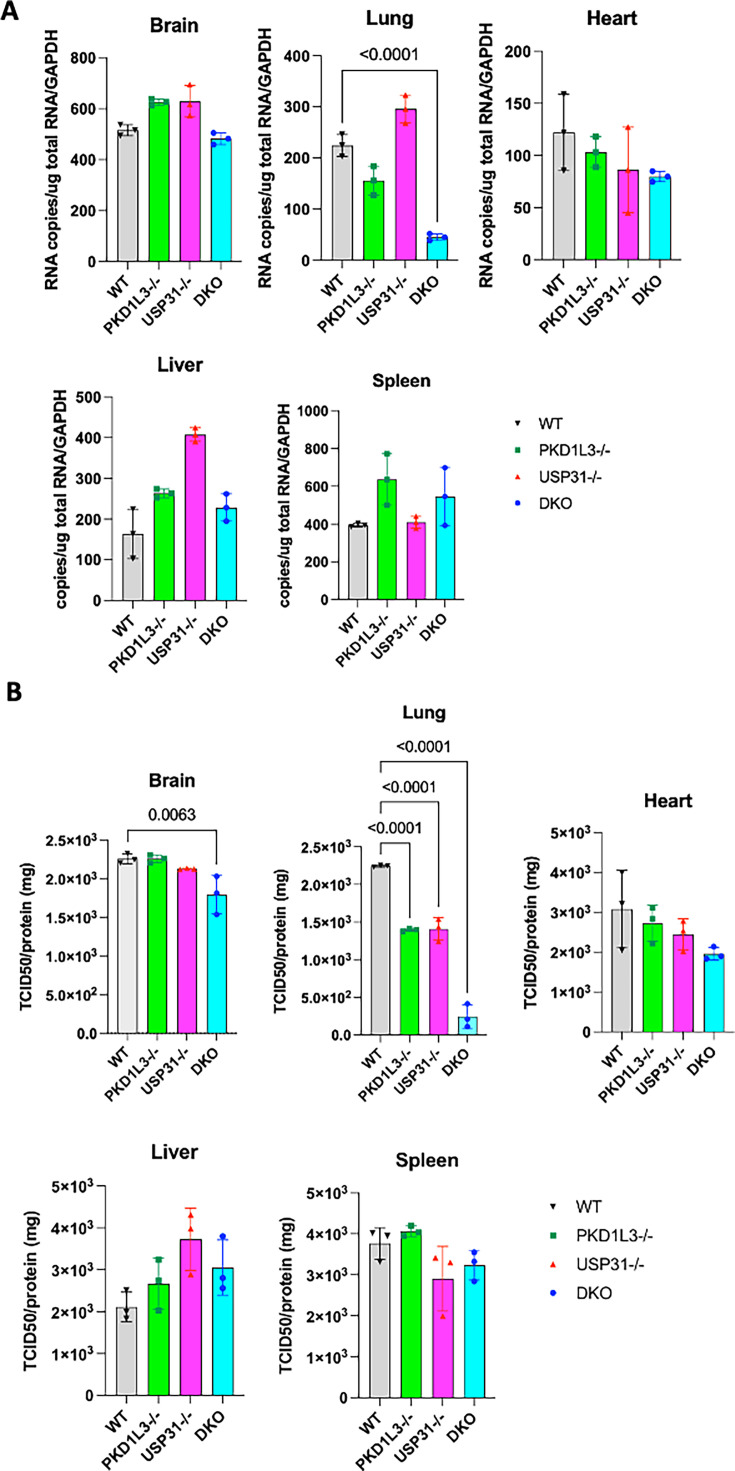
EMCV RNA amounts (copies/μg total RNA/GAPDH) (**A**) or viral titer (TCID_50_/mg total protein) (B) in brains, spleens, livers, lungs, and hearts from WT *Pkd1l3*^−/−^, *Usp*31^−/−^, and DKO mice (*n* = 3). A *P*-value of less than 0.05 is indicated as statistically significant.

EMCV RNA was significantly reduced in the lungs of double-knockout mice versus WT mice (*P* = 0.0116), but showed no other significant differences between genotypes in any other organ, although the copy numbers showed relative decreases in the brain and heart of DKO mice (Fig. 6A). The EMCV viral titer in each target tissue was measured, and the results (Fig. 6B) showed a broadly similar tendency with significant reductions observed in lung tissue in all KO mice, and in brain tissue in the DKO mice.

## DISCUSSION

In the current study, we aimed to establish single- and double-knockout mice to investigate the deletion of host factors implicated in IRES-mediated translation for FMDV and CSFV, and evaluate the effects on IRES activity and virus infection in the relevant (*Pkd1l3*^−/−^, *Usp31*^−/−^, and DKO) mice. To the authors’ knowledge, this is the first such *in vivo* evaluation of single and double *Pkd1l3*^−/−^ and *Usp31*^−/−^ knockdown *in vivo*. As key findings in this study, we established knockout mice as a viable test system for this evaluation and demonstrated the contributions of the relevant host factor deletions to conferring resistance to viral infection. With a genetic engineering approach, we were able to knock out the CD163 viral receptor. This receptor knockout is essential for establishing porcine reproductive respiratory syndrome virus (PRRSV)-resistant pigs, and should mitigate the damage induced by viral infection (4, 15). Our study highlights the powerful potential utility of a gene knockout for establishing disease-resistant livestock.

*Pkd1l3*^−/−^ mice showed no significant differences in body weight or survival relative to WT mice, and no sex bias in the number of births; these findings are consistent with a previous study in mice lacking this gene (5). The *Usp31*^−/−^ and *DKO* mice showed lower body weight than WT in adult mice, and DKO mice showed significantly lower body weight gain than WT during 21–28 days after birth. We speculate these lower body weights reflect the absence of the NF-κB enhancement activity when *Usp31* is deleted, as previously reported (6). Nevertheless, mice in three genotypes showed positive body weight gain and were viable through 1 year after birth, as well as showing a biologically plausible F1 genotype distribution (not marked deviation from true Mendelian inheritance).

PKD1L3 is reportedly expressed in taste cells and forms heteromers with PKD2L1 that function as sour taste receptors (7). Quantitative RT-PCR revealed higher PKD1L3 expression in the liver and testis than in the kidney, and northern blot analysis revealed a 6.0-kb transcript with maximal expression in the placenta and minimal expression in the heart and lung (16). USP31 belongs to a large family of cysteine proteases that function as deubiquitinating enzymes (17). RT-PCR and ELISA showed ubiquitous expression of USP31 in adult and fetal human tissues (8). Northern blot analysis of human tissues revealed expression of a 5.0-kb transcript only in the testis. In this study, deletion of PKD1L3 and USP31 did not significantly influence F1 genotype distribution, possibly because of these proteins’ restricted tissue distribution and their role in maintaining vital functions. Examination of viral replication in tissues with abundant expression of these proteins, such as sour taste cells in knockout mice, should provide some insight into their role in viral replication. To account for the influence of viral genome mutations during infection, we need to characterize the relevant genome using direct sequencing or next-generation sequencing (18).

Our evaluations of IRES activity in the liver revealed two tendencies. First, FMDV activity showed stronger—and significant—suppression across mice of all three knockout genotypes, whereas the reductions in CSFV IRES activity versus the activity in WT mice were not significant. These *in vivo* findings are consistent with those in our previous *in vitro* study using FMDV and CSFV-IRES bicistronic RNA, in which we found that silencing of *Pkd1l3* alone and both *Pkd1l3* and *Usp31* suppressed IRES activities, and silencing of *Usp31* had a suppressive effect on CSFV IRES activity, but of lesser magnitude (9). We also found stronger suppression of IRES activity in *Pkd1l3^−/−^* mice than either *Usp31*^−/−^ or DKO mice, and these findings are also consistent with the results of our previous *in vitro* studies (9, 19). This may suggest that PKD1L3 plays a more significant role in the regulation of IRES activity than viral replication. We observed possible associations between PKD1L3 and the canonical translation factor eIF3c (20) and the chaperone heat shock protein (HSP)90β (21), both of which have been implicated in hepatitis C virus IRES-mediated translation (22). Further insights into the regulatory role of PKD1L3 in IRES activity may be gained from investigation of possible roles in the regulation of calcium concentration for PKD1L3/PKD2L1 (7).

Our findings on CSFV replication present something of a contrast to those for IRES activities. We found stronger suppression of replication in U*sp31*^−/−^ and DKO mice than *Pkd1l3*^−/−^ mice, and the magnitude of the suppressive effect was strongest in DKO mice. USP31 is a deubiquitinating enzyme, and its overexpression is known to suppress TNFα-mediated NF-κB activation (23). Therefore, we consider that the deletion of USP31 may contribute to inhibition of CSFV replication through NF-κB activation by inducing expression of type I interferon (IFN) (24) or type III IFN (IFNλ) (25). Furthermore, the deletion of *USP31* may contribute to the inhibition of EMDV replication through NF-κB activation and the resulting type I IFN production (26). Thus, knockout of *Usp31* may suppress viral replication through activation of NF-κB signaling and further suppress viral replication by inhibition of IRES activity through silencing of *Pkd1l3*, as demonstrated by the maximal inhibitory effect on CSFV replication in DKO mice.

We also evaluated mice of three genotypes after infection with EMCV (as a surrogate for FMDV based on similarity in IRES structure), based on histopathological assessment and virus quantification in organs and tissues. Histopathologically, WT mice showed a range of lesions consistent with disease, including necrosis in the cerebral cortex, which has previously been reported in mice experimentally infected with EMCV (3), and the overall presentation was consistent with reports of milder pathology in EMCV-infected C57BL6/J mice (27). In quantification of virus level, the decreased RNA copy number in DKO mice was the only significant difference with WT mice (no significant differences were observed in the heart, brain, spleen, or liver in *Pkd1l3*^−/−^, *Usp31*^−/−^, and DKO mice). The effect of gene knockout on EMCV infection was most pronounced in the lung, where all KO mice showed significant reductions in viral titer, while the DKO mice showed the most significant reductions in the brain. This may reflect the stronger effect of KO at the translation level during EMCV infection. Tissues were collected at day 7 after inoculation, and EMCV infection reportedly peaks between day 3 and day 7 in C57BL6/J mice (10). Accordingly, we suggest that future studies with tissue collection points across this period may yield significant results. The difference in the lung may reflect stronger NF-κB activity there, as a site where immediate immune responses to foreign substances are mounted due to constant contact with elements of the external environment (28, 29). Accordingly, we consider that NF-κB activation in the absence of USP31 may be stronger in the lung than in other organs and tissues, and that leads to a further significant suppressive effect on EMCV infection in the lung tissue of DKO mice.

Despite the new insights it provided, this study possesses several limitations. First, the effects of PKD1L3 and USP31 knockout on FMDV and CSFV infection could not be fully evaluated in our KO mice, because of restrictions imposed on this type of research for biosecurity reasons under Japanese law, and the fact that mice are largely unsusceptible to CSFV infection. Second, the influence of PKD1L3 and USP31 silencing was not fully addressed, and we cannot exclude the possibility of unintended physiological consequences emerging in livestock offspring. Transient inhibition of *Pkd1l3* and *Usp31* may represent an alternative strategy that could prevent unintended physiological consequences that might result from the knockout of these two genes.

In conclusion, this study provides evidence that knockout of genes encoding host factors implicated in IRES-mediated translation (*Pkd1l3* and *Usp31*) may provide some protection against infection with FMDV and CSFV *in vivo*. The suppressive effects against viral replication were highest in lung tissues in DKO mice; furthermore, these mice appeared normal clinically and viable through 1 year after birth, suggesting mice are promising candidates for further *in vivo* evaluations of these host factor deletions. Accordingly, we suggest that our results for DKO mice support the potential for multi-disease-resistant livestock to act as a mitigation strategy for FMDV and CSFV infection. However, further research should concentrate on detailed elucidation of DKO mouse phenotypes and characterization of the mechanisms by which IRES-mediated translation and replication are suppressed. Although the host factors through which PKD1L3 and USP31 influence viral replication have not yet been identified, eIF3c and HSP90β may mediate their effects on translation (9). Further characterization of these transport-regulating networks should be addressed in a future study.

## MATERIALS AND METHODS

### Mice

C57BL6/J mice heterozygous for the *Pkd1l3* or *Usp31* gene were obtained from Cyagen Biosciences (Japan) Inc. (Osaka, Japan). Specifically, the mice were harboring a heterozygous *Pkd1l3* or *Usp31* knockout (*Pkd1l3^+/−^* or *Usp31^+/−^*) generated by Crispr/Cas9-mediated genome editing, which had been commissioned to Cyagen Biosciences before delivery to our laboratory. For the knockout of the *Pkd1l3* gene, the following guide RNAs (gRNAs) were designed to delete exons 2–5 (Fig. 1A): A1 (5′-TGTGCACCATCATTGACCGGTGG-3′, forward strand), A2 (5′-AGCCTGCCCCAATGTACCAGAGG-3′, forward strand), B1 (5′-AATTCCTGAGTCTTCATGTATGG-3′, forward strand), and B2 (5′-CAATAGGCACTCAACCCACTGGG-3′, reverse strand). For the knockout of the *Usp31* gene targeting, the following gRNAs were designed to delete exons 2–9 (Fig. 1B):

A1 (5′-TACCGGTGTGTTTAGAACACAGG-3′, forward strand),

A2 (5′-CTCATGTAGAAATTAAGTACTGG-3′, forward strand),

B1 (5′-GTTTCACTGATACATAGTGAAGG-3′, forward strand),

B2 (5′-AAGCCCTCCCAACTTACTTTTGG-3′, reverse strand).

The mice were housed under a 12-h light (7:00–19:00)/dark cycle at 22°C ± 2°C with *ad libitum* access to chow and water at the Transboundary Animal Diseases Center, Joint Faculty of Veterinary Medicine, Kagoshima University, Japan.

Heterozygous knockout mice were intercrossed to generate homozygous single-knockout offspring (knockout for each host factor).

### Genotyping of F1 mice

Genomic DNA was isolated for PCR-based genotyping with the following procedure: A small tissue sample was collected from the tail or ear of each mouse, and immersed in 50 mM NaOH 150 μL 95°C for 10 min, after which 1M Tris-HCl, pH 8.0 (20 μL), was added. The resultant mixture was centrifuged (12,000 rpm, 5 min). The supernatant was collected for amplification using Platinum II Hot-start PCR master mix (ThermoFisher Scientific) at 94°C for 2 min, denaturation at 94°C for 15 s, annealing at 60°C for 15 s, and extension at 68°C for 15 s/kb) for 35 cycles. PCR products were resolved by agarose gel electrophoresis, stained with ethidium bromide, and visualized as bands using Fusion Solo (M&S Instrument Co.). For *Pkd1l3* genotyping, F2 and R1 primers amplified 734 base-pair (bp) DNA fragments for WT and F1, and R1 primers amplified 564-bp fragments for the single homozygous knockout (Fig. 1C). For *Usp31* genotyping, F1 and R2 amplified 597-bp fragments for WT and F1 and R1 primers amplified an 800-bp fragment for the single homozygous knockout (Fig. 1D). Primer sequences were as follows:

*PKD1L3* F1 5′-AGGGTTAGTTTGACCATCAATCTCT-3′

*PKD1L3* F2 5′-AGAGTTCAACCAGTCAGGTAAGTC-3′

*PKD1L3* R1 5′-TCACCATGGGAGTAGAGTAAATGG-3′

*USP31* F1 5′-GTGGTGATATAGGATGGGCTTGAT-3′

*USP31* R1 5′-GGATGAGTAGAGTTCAAACTATGGGG-3′

*USP31* R2 5′-GAGGAATTCTCCTTCAACAGGCTA-3′

The positions of these primers on the mouse genome are indicated in Fig. 1A and B.

For genotyping PCR and RT-PCR, 1.5% agarose gel was utilized and 1 kb DNA Ladder plus (NIPPON Genetics Co.) was used as a marker.

### Detection of Pkd1l3 and Usp31 mRNA by RT-PCR

Total RNA was extracted from mouse liver tissues using ISOGEN (NIPPON GENE, Tokyo, Japan) as previously described (30) and treated with RNase-free DNase (Promega) following the manufacturer’s protocol. Complementary DNA (cDNA) was reverse transcribed from these RNAs using Superscript III (ThermoFisher Scientific) following the manufacturer’s protocol. *Pkd1l3* gene was amplified using *PKD1L3* RTF1 and RTR1 primers, which yielded DNA fragments (242 bp) only for WT mice and not for the homozygous knockout mice. For *Usp31* genotyping, amplification was performed using RTF1 and RTR1 primers, which yielded DNA fragments (361 bp) only for WT mice and not for the homozygous knockout mice.

*PKD1L3* RTF1 5′-CCTCAAGGTGCAAGCATTTGG-3′

*PKD1L3* RTR1 5′-TGTGGCCTTCCAGTTAGGGA-3′

*USP31* RTF1 5′-TGGGACTGTTGCCAGACTTC-3′

*USP31* RTR1 5′-GGCCCGGTTACATACAAGCA-3′

The positions of these primers on the mouse genome are indicated in Fig. 1A and B.

### *In vivo* reporter assays

IRES activities were evaluated in mouse tissue, using a bicistronic pRF plasmid for the FMDV IRES as described previously (31), a bicistronic pCI5 plasmid for the CSFV IRES, as described previously (12), and an EMCV IRES-expressing bicistronic vector (pRE-IRES) (32). Plasmid DNA (40 μg) was mixed with *in vivo* jetPEI^R^ (Polyplus-transfection SA, Illkirch, France) 6 mL in 5% glucose (200 mL), and this mixture was injected retro-orbitally, using a 27G needle over a 2-s period (33).

At 24 h post-injection, mice were sacrificed by exsanguination under anesthesia induced by ketamine (5 mg/mL) and xylazine (0.5 mg/mL) in PBS (100 μL/mouse) and maintained with isoflurane (2%) aspiration. The lungs, liver, heart, kidneys, and spleen were collected from each mouse and evaluated comparatively to identify the tissue with the maximal incorporation efficiency for direct measurement of luciferase activity.

Tissues were lysed with passive lysis buffer (PLB), and luciferase activity was measured using a dual-gro luciferase assay kit (Promega). IRES activity was calculated as the yield ratio of F-Luc/R-luc activities measured by Envision (PerkinElmer Co.).

Replication was also evaluated in mouse tissue for CSFV using a replicon RNA (Fig. 3B). The replicon RNA was synthesized from a vCSFV GPE^−^/HiBiT recombinant classical swine fever virus encoding the HiBiT luciferase gene (rGPE-N^pro^-Luc-IRES-NS3) as described (34), which is derived from recombinant full-length cDNA of the CSFV GPE^−^ strain (35). The CSFV replicon RNA (60 μg/mouse) was retro-orbitally injected into mice using *in vivo* jetPEI (Polyplus-transfection SA, Illkirch, France) to characterize the effect on CSFV replication by using a Nano-Glo HiBiT lytic detection system (Promega, Madison, WI, USA), as described previously (34). The mice were sacrificed at 24 h post-inoculation, and tissues were collected for evaluation in the same way as described for the IRES activity reporter assays described above. The protein concentration in each lysate was measured using the micro-BCA protein assay kit (Thermo Fisher Scientific Co.). RNA was isolated using ISOGEN (NIPPON GENE Co.) and RNeasy Mini Kit Plus (Qiagen Co.), and GAPDH mRNAs were then measured using mouse GAPDH endogenous control (ThermoFisher Scientific Co.). FLU activity in each mouse liver lysate was measured and normalized to protein concentration (μg/mL) and GAPDH copy number (copies/mL) to determine CSFV replication.

### Infection experiment

Mice were infected with EMCV (purchased from ATCC) by intraperitoneal injection (4 × 10^5^ TCID_50_) (3). The mice were sacrificed at 1 week post-inoculation as described for the *in vivo* reporter assays (above), and organs and tissues were collected from each mouse. Specimens for histopathological examination were prepared from the brain, spleen, liver, lungs, and heart, and evaluated after staining with hematoxylin and eosin (H&E) stain and observed as previously described (36).

Total RNA was extracted and assessed as described above, and to determine RNA levels in WT, both single-knockout and double-knockout mice. EMCV RNA levels were quantified using FAST SYBR Green QRT-PCR Master Mix (Agilent Technologies) and primers 241S (5′- AACGTCTGTAGCGACCCTTT-3′) and 409A (TACGCTTGAGGAGAGCCATT-3′) (9), which are designed within EMCV-IRES sequences, and calculated as copies per microgram of total RNA, normalized to GAPDH, in brain, spleen, liver, lung, and heart tissues from WT*, Pkd1l3*^−/−^, *Usp31*^−/−^, and DKO mice. *P* < 0.05 was considered statistically significant and is indicated. The viral titer in each target tissue was determined after homogenization with Leibovitz’s L-15 medium using a homogenizer (Bio macher II, Nippi Co.) and centrifugation at 5,000 rpm for 5 min. The protein concentration of the obtained supernatant was measured using a Micro BCA protein assay kit (ThermoFisher), and the supernatant was serially diluted 10-fold for mixing with BHK cells on a 96-well plate. The viral titer (TCID_50_) was calculated using the Behrens-Kärber method.

### Statistical analysis

IRES activities, RNA expression levels, and body weight were compared between genotypes. All data are presented as mean ± standard deviation (S.D.) from three independent experiments, and figures were generated using the GraphPad PRISM (version 9) software. Statistical analysis was performed using one-way ANOVA and Dunnett’s test, or Student’s *t-*test to evaluate significant differences. *P* values < 0.05 were considered significant.

## Data Availability

The results in this paper are available upon reasonable request to the corresponding author.
